# Supplementary material for: Are carbon emissions trading and green financial instruments synergistic? -Comprehensive quantitative research based on content analysis
Source: PLoS One. 2024 Mar 7;19(3):e0298601. doi: 10.1371/journal.pone.0298601 (PMC10919601; doi:10.1371/journal.pone.0298601)
Supplement: S1 Data — (ZIP) [file pone.0298601.s001.zip › Data/地级市Z_F工作报告.zip!/╜¡╬≈/─╧▓2╩╨/└┤╫╘╣1⁄2╓┌║┼í╛┬φ┐╦╩2╛▌═°í┐_2010.docx]

各位代表：

　 现在，我代表市人民政府，向大会作政府工作报告，请予审议，并请各位政协委员和列席会议的同志提出意见。

　 一、2009年工作简要回顾

　 过去的一年，是新世纪以来我国经济发展最为困难的一年，也是我们各项工作取得显著成效的一年。在省委、省政府和市委的坚强领导下，全市上下全面贯彻胡锦涛总书记“三保一弘扬”的重要指示精神，深入学习实践科学发展观，认真落实中央和省扩大投资、拉动内需的方针政策，主动应对，攻坚克难，大力实施新一轮经济大发展、城乡大变样、文明大提升“三大工程”，实现了主要经济指标增幅在全国省会城市中位次前移，经济总量占全省比重有所提高，经济发展态势总体回升向好。全市预计实现地区生产总值1820亿元，增长13%；全社会固定资产投资1559亿元，增长41.9%；社会消费品零售总额634亿元，增长20%；城镇居民年人均可支配收入16472元，增长9%；农民年人均纯收入6294元，增长9%；人口出生率13.86‰；城镇登记失业率控制在3.41%。上述指标均完成了市人大十三届四次会议确定的目标任务。其中全社会固定资产投资、社会消费品零售总额、城镇居民年人均可支配收入、农民年人均纯收入提前一年完成“十一五”规划目标。由于国际金融危机的冲击及国家支持企业减免税费政策的出台，财政总收入249.77亿元，增长11.5%；地方一般预算收入115.83亿元，增长13.4%；外贸出口21.3亿美元，下降14.9%，这三个指标未能达到预期目标。市政府已按有关法定程序向市人大常委会作了说明。回顾去年以来政府工作，主要抓了以下六个方面：

　 （一）科学应对金融危机，经济平稳较快增长

　 工业经济稳中有升。坚持上项目扩投资、保企业促发展、调结构强产业。出台扶持企业发展“50条”、促进开发区做强做大“26条”等政策，整合10亿元专项资金支持企业发展，通过增值税转型改革为企业直接减免税收10亿元。“3010”工程和“十百千亿工程”进展顺利，新昌电厂一期、赛维BEST太阳能薄膜电池一期、南钢技改一期、海立电器、人民电器等一批重大项目竣工，南昌航空工业城、实德（安义）型材园等一批重大项目开工，太阳能光伏、LED照明、新能源汽车、生物医药、绿色食品等产业集聚效应初步显现。高新区、经开区、昌东工业区、小蓝经济开发区主营业务收入分别达到520亿元、310亿元、250亿元和210亿元。全市工业实现主营业务收入迈上2000亿元新台阶，达2040亿元，增长14%；规模以上工业增加值615亿元，增长18%；企业经济效益综合指数260%，较上年提高11个百分点。

　 农业生产巩固提高。落实强农惠农政策，发放种粮综合直补、农机具购置补贴4.54亿元。大力实施造地增粮富民工程，新增耕地7292亩，全年粮食总产量22.9亿公斤，增长1.9%；生猪出栏324万头，增长10.9%；水产品起水量34万吨，增长5.8%。实施农业标准化战略，扎实抓好“双十双百双千” 工程、“一村一品”、村干部“双带致富”工程。整合涉农资金和项目，推进农业产业化进程，已有国家级龙头企业9家、省级龙头企业44家、市级龙头企业186家。国旺、南昌菜园等18家蔬菜基地年产蔬菜151万吨。蒋巷现代农业示范园、黄马“两江”生态农业走廊、安义现代农业科技园对现代农业示范作用凸显。

　 现代服务业发展加快。被评为“中国服务外包示范城市”，引进贝塔斯曼、用友红谷滩产业基地、泰豪国际动漫产业园等一批软件和服务外包项目。新增5个超万平方米商场，老城区升级改造11家集贸市场，新建改造912家“农家店”，设立“家电下乡”销售网点674家，发展农村现代流通网点3250个；深圳农产品批发市场等2个项目被列为全国“双百市场工程”项目；江西煌上煌集团等4个项目被列为“农超对接”项目。南昌保税物流中心（B型）通过省验收。全市金融机构年末存款余额3289.88亿元，比年初增长31.6%；贷款余额2932.12亿元，比年初增长36.1%。大新银行、民生银行、洪都农村商业银行、九江银行开业，光大银行、赣州银行正式落户，成功举办“首届中国南昌金融展”。出台《加快旅游产业大市建设的若干意见》，天香园二期、宝葫芦农庄等一批旅游景区景点加快建设，国内外游客人数和旅游业总收入持续增加。成功举办第38届全国制药机械博览会、中国绿色食品博览会、第四届中国南昌国际汽车展等全国品牌展会，连续四年荣获中国十佳会展城市称号。

　 （二）改革开放不断深化，发展活力显著增强

　　 国有工业企业改革基本完成。认真落实省委、省政府提出的国有企业改革的指导原则，坚持以人为本、依法操作、精心部署、稳妥推进，充分发挥企业班子和工会的作用，切实维护广大职工的合法权益，全市68户国有工业企业（含12户省属下放企业）改制工作进展顺利，分流安置职工12万人，化解了多年来历史遗留问题，推动了市属国有工业企业资产整合、机制转变、靠大联强、裂变扩张。

　 各项改革稳步推进。稳步实施市、县政府机构改革和事业单位人事制度改革，启动实施义务教育学校绩效工资改革。深化投融资体制改革，各级融资平台建设进展顺利，企业债券发行工作加快推进；确定了以国开行为牵头行的银团，为轨道交通1、2号线提供融资贷款。扎实推进医药卫生体制改革。加大文化体制改革力度，启动专业艺术表演团体改革。

招商引资力度加大。大力推进产业招商，承办首届世界低碳生态经济大会暨技术博览会、中国“五会”(南昌)经贸恳谈会、第七届赣台经贸洽谈会，主办第八届中国南昌金秋经贸活动月等活动。组织招商小分队推介招商。全年实际利用外资15.8亿美元，增长12%；实际利用内资453.2亿元，增长13.94%。全年新批外商投资项目145个，其中超千万美元项目43个；新批内资项目767个，其中超亿元项目55个。目前，已有36家世界500强企业、37家国内200强企业落户南昌。

　 对外合作不断拓展。开通南昌—台北直航航线，扩大昌台开放交流。与德国派尼区缔结友好城市，加强与东盟各国的合作交流。巩固“泛珠三角”省会城市合作平台，与南京、合肥携手打造“宁合昌”新三角合作，与宁波结为友好城市。连续第6年被《福布斯》评为中国大陆最佳商业城市、被台湾电电工会评为台商投资“极力推荐城市”。

　 （三）城市功能不断完善，城乡面貌进一步改观

　 规划龙头作用进一步增强。落实土地规划修编政策，加快了市县土地规划修编工作，新增建设用地指标规模。完成城东、城南、城北地区及红谷滩凤凰洲等片区控制性详规；完成老城区重要堵点交通改善、“七城会”期间交通组织实施、西客站地区等综合交通规划；完成宾馆设施布局、玉带河景观、城市发展战略等专项规划；《南昌港总体规划》获省部批准；推动县城总体规划及控制性详细规划、重要地段修建性详规的编制工作。强化规划严肃性，严格审批建设项目、审查规划建筑方案、审议交通影响评价报告，加强规划土地执法管理。

　 基础设施建设进一步推进。政府主导投资推进城建项目42项，总投资532亿元。英雄大桥、青山湖隧道竣工通车；阳明路综合整治及八一大桥维修加固、高新南大道三期、紫阳大道综合改造等工程完成；昌九城际铁路全线铺轨，昌北机场新航站楼封顶封围，南昌枢纽西环线通车。南昌轨道交通项目经国家批准建设。轨道交通1号线一期、青山湖大道、广州路改造、瑶湖大桥扩建及麻丘互通立交、朝阳片区11条主次干道等工程开工；生米大桥西连接线西延工程、艾溪湖大桥、三店西路立交等项目前期工作进展顺利。新昌电厂首台机组投产送电，天然气管网一期工程昌北地区实现通气。青山湖污水处理厂17万吨扩容工程竣工，象湖、红谷滩污水处理厂收集管网及泵站工程顺利完工；四县、桑海开发区污水处理厂建成投运。南昌国际体育中心土建工程封顶，江西前湖迎宾馆完成主体工程。

　 城市管理水平进一步提升。实行建管分离，组建市城市管理委员会。开通城市管理12319热线，开辟城市管理专栏；建立实行“日巡查、周调度、月排名、季考核、年奖励”的城市管理考核机制，推进数字化综合城市管理体系建设。实施八一大道、赣江两岸景观亮化工程，完成16条道路店招整治和25个立面节点改造工程。新建25个市政、园林取水点；升级改造48座垃圾中转站，更新垃圾运输车，新建一批公厕、交通护栏、路口交通信号灯。

　 农村面貌进一步改观。坚持统筹城乡发展，完成652个新农村自然村点整治建设，改水2.96万户，改厕2.92万户，整修房屋63.6万平方米，建设沼气池1797户，完成380个自然村广播电视“村村通工程”。按照“户集、村收、乡镇运、县区处理”的模式和流程，推进农村垃圾无害化处理。推进农村信息化工作，成为全国19个农村信息化试点城市之一。完成自然村公路硬化1000公里；29个渡改桥项目开工建设26个、竣工5个；24座国家专项规划内的水库除险加固工程竣工15座；12个国家中部四省规划的大型泵站更新改造工程竣工8个；65座小型水库坝下涵管实施改造；启动鄱阳湖治理二期扬子洲圩堤工程建设。

　 生态人居环境进一步改善。继续实施城区水系活化工程，玉带河综合整治工程五干一分渠、北支引水活化工程顺利实施，启动乌沙河综合整治。启动 “森林城乡、花园南昌”建设，完成造林面积46.9万亩，被评为全省造林绿化“一大四小”工程建设先进市。城市绿化覆盖率41.92%，绿地率39.43%，人均公园绿地8.46平方米。强力推进污染减排攻坚战，城市环境综合整治定量考核连续5年排全省前列；万元生产总值能耗下降4.5%；二氧化硫提前完成“十一五”减排任务。空气质量优良率95.1%；集中式饮用水源水质达标率100%。以“鄱湖清水美”荣获中国十大最美丽城市称号。

　 （四）坚持关注改善民生，社会事业全面进步

　 民生工程扎实推进。投入专项资金29亿元，其中省财政补助15.9亿元、市本级投入8.5亿元，县区财政投入4.6亿元，民生工程八个方面、62件实事全面落实。新增城镇就业9.4万人，“零就业家庭”安置率100%；新增转移农村劳动力3.7万人，农村劳动力转移培训4.75万人。统筹范围内离退休人员养老金100%按时足额发放。城区低保标准由每人每月230元提高到300元（四县及桑海开发区从190元提高到260元），农村由80元提高到120元。农村五保集中供养率80%。城镇职工参加养老保险104.9万人；城镇居民基本医疗保险61.26万人。新型农村合作医疗参合率97.07%，提高住院补偿线标准。医疗救助1.8万人次。解决22.46万农村人口饮水安全问题。落实计划生育奖励扶助对象3727人，办理计划生育家庭爱心保险9.44万人。建设农家书屋137家。对66.24万名公办、民办义务教育阶段学生免除学杂费、免费提供教科书和地方通用教材；对义务教育阶段困难家庭寄宿学生、困难家庭高中生及大学新生发放资助金2701万元；“春蕾助学行动”顺利实施。儿童国家免疫规划疫苗免费接种覆盖面100%，五苗常规接种率和新增疫苗接种率90%以上。对妇女儿童、残疾人、外来民工等群体专设法律援助工作站，办理法律援助案件2000余件。经济适用住房实物住房建设1410套、货币补贴3300套落实到位。廉租住房4537套任务中有3637套主体完工。

　 各项事业协调发展。深入开展文明创建活动，2009年公共文明指数测评列全国省会、副省级城市第11位。编制中心城区中小学网点布局规划，推动农村中小学校舍新建改造及配套完善工作，青云谱实验学校、东湖阳明学校、朝阳新城云飞路学校、南钢学校等7所九年一贯制示范学校加快建设，启动南昌一中朝阳新城校区、南昌十九中红角洲校区建设，师大附中红谷滩滨江分校建成招生。新增省重点中学13所、省重点建设中学6所。坚持科技强市，被列入国家“十城千辆”、“十城万盏”工程试点城市；专利申请量1802件、授权量1023件，分别比上年增长25.31％、17.72％。18项新产品获省科技创新基金支持，占全省56%。新认定市级以上高新技术企业21家。获省级科技进步奖12项。连续八年获全国科技进步先进市称号。评选了一批“洪城特聘专家”、“211”企业经营管理人才、“市优秀农村实用人才”，9人获国家级、省级“百千万人才工程”奖。推进县级医疗机构国债项目建设，完成中心卫生院建设5家、村卫生所230个、标准化社区卫生机构218个。推行医疗机构网上药品招标采购。扎实开展“光明·微笑”行动，完成白内障手术治疗13609例、唇腭裂手术治疗660例。扎实推进公共卫生防疫体系建设，科学应对、有效防止甲型H1N1流感疫情扩散。完成了人口计划，出生人口性别比连续三年下降。完成《南昌市公共文化设施布局规划》，八大山人真迹陈列馆、西湖市民文化广场等一批重大文化场馆开工建设。大型采茶戏《一家人》正式公演，广播剧《回家》获中国广播电视政府奖二等奖、专家金奖。有线电视数字转换率90%以上。举办“重走火炬路、圣火耀神州”、龙舟邀请赛等群众体育活动，获全国群众体育先进单位；有序开展第十三届省运会备战工作；扎实推进“七城会”各项筹备工作。

　 （五）狠抓平安南昌创建，保持社会和谐稳定

　 以庆祝建国60周年等重大活动为重点，以营造安全和谐的社会环境为目标，启动“法治南昌”创建，全面推进法治县区、单位（部门）和民主法治村（社区）建设。司法所建设成效明显。坚持科技强警，实施“天网”工程，建立群防群治“十支队伍”，开展“十项防范”活动，加强社会治安综合治理，深入开展打黑除恶、命案侦破、打击“两抢一盗”、非法河道采砂和文化市场“扫黄打非”斗争等专项行动，严厉打击各类违法犯罪活动。切实加强交通安全、生产安全、消防安全、食品药品安全、森林防火安全，获2009年全国安全生产先进单位。高度重视信访工作，严格实施领导“一岗双责”、领导包案和市政府领导信访值班制度，抓好市长“手机、信箱、热线”反映问题的落实，关注民生、化解矛盾。进一步完善应急预案，健全应急队伍，不断提升应急处置能力。全市没有发生大规模群体事件、严重暴力性事件、重大治安事件、恶性火灾事件和重特大生产经营性事故，荣膺“2009中国十大最具幸福感城市”。

　 （六）加强作风效能建设，扎实推进政府工作

深入开展学习实践科学发展观活动，落实领导干部学法用法制度。开展“机关效能年”活动，加快市、县（区）行政服务中心、乡镇便民惠民服务中心及公共资源交易中心、交易站建设。扎实推进行政审批“两集中三到位”，完成“三个压缩30%”任务。加大立法工作和执法监督力度，全年提请市人大常委会审议地方性法规草案5件，制定和修改政府规章5件，审查报备规范性文件59件；落实行政执法责任制，规范行政执法行为和行政处罚自由裁量权，受理行政复议案件109件。定期向人大报告工作、向政协和社会通报情况，建立人大代表建议、政协提案交办、督办和办结制度，688件人大代表建议和政协提案全部办理完毕，代表委员满意率不断提高。坚持政务公开，通过召开听证会、公开展示、网上征求意见等方式，依托公共图书馆、档案馆和行政服务中心建设政府信息查阅场所，坚持重大事项广泛听取各方意见，提高科学民主决策水平。抓好惩治和预防腐败体系建设，全面落实党风廉政建设责任制，严格执行领导干部廉洁从政各项规定，严肃查办违纪违法案件。加大纠风治乱和效能监察力度，认真治理损害群众利益的突出问题。

2009年，我们还成功举办系列规格高、规模大、影响广的重大活动，展示了南昌的形象和品牌，提升了知名度和美誉度。一是承办了世界首届低碳生态经济大会暨技术博览会。来自世界500强企业、国内200强企业、台湾100大企业，25个外国驻华使领馆使节、国际机构代表，著名院校和科研院所专家学者等国内外重要嘉宾近1400人，围绕“节能减排·绿色生态”的主题，举办了19场重大活动，开展了广泛的研究探讨，签订38个重大重点项目。二是承办了第18届中国金鸡百花电影节。中国电影界的知名人士齐聚南昌，举办了“与新中国同行”、明星走秀和颁奖典礼等诸多活动。三是主办了夏娜南昌国际艺术动漫周。法国、巴西等40多个国家和地区以及上海、浙江、江苏等国内动漫产业潮流地区的企业和基地组团参加，举办了高峰论坛、欧洲电子视频音乐表演等系列活动。四是主办了第三届中国·南昌国际军乐节。来自美国、英国、俄罗斯等国家及地区和中国人民解放军、海军等知名军乐团，围绕“大气开放、响亮南昌”的主题，举办了军乐游行和七场晚会、音乐会。五是承办了全国农业综合开发工作会议。向全国充分展示了我市近年来农业综合开发所取得的丰硕成果，受到了各级领导高度肯定。

与此同时，大力开展国防教育，加强人防建设，推进优抚安置和双拥工作；外事侨务工作成绩显著，被评为全国侨务工作先进集体；民族宗教、对台事务工作得到加强；统计、审计、工商、物资、参事、档案、地方志、保密、老龄、妇女儿童、残疾人（出台了《南昌市关于促进残疾人事业发展的实施意见》）、消费者权益保护、防震减灾、科学普及、质量技术监督等工作，都取得了新的成绩。

　 各位代表，过去的一年，南昌经济社会发展在极为困难的形势下取得如此成绩，实为不易。这是全市人民在市委的正确领导下，认真贯彻党中央、国务院的大政方针和省委、省政府的战略决策，团结拼搏、务实创新的结果；是市人大依法监督和市政协民主监督的结果；是各民主党派、工商联、无党派人士、各人民团体和驻昌单位、人民解放军、武警官兵以及离退休老同志共同努力、大力支持的结果。在此，我谨代表市人民政府，向全市人民，向所有关心、支持南昌发展的同志们、朋友们，表示衷心的感谢并致以崇高的敬意！

然而，我们也清醒地看到，与沿海发达城市和中部省会城市相比，与省委、省政府和全市人民对我们的殷切期望相比，我市的经济总量还不大，综合竞争力还不强。主要表现在：增加就业岗位、增加财政收入的支柱产业、优强企业还不够多，产业集聚和辐射力还不强；城市功能仍不够完善、城乡科学管理水平有待提高，统筹城乡发展步伐亟需加快；就业、教育、医疗、住房、出行等群众关心的重点问题仍有大量工作要做；节能减排、环境保护、维护稳定的任务仍然繁重；政府效能有待进一步提升。对此，我们将采取切实有效措施，认真加以解决。

　 二、2010年工作安排

　 今年是实施“十一五”规划的最后一年，也是全面实施鄱阳湖生态经济区建设的起始之年。做好今年的工作，对有效应对国际金融危机冲击，保持南昌经济社会又好又快发展，圆满完成“十一五”规划、顺利启动实施“十二五”规划，意义十分重大。

　 当前，国际金融危机的深层次影响依然存在，国内经济发展态势明显企稳向好，但经济增长的内生动力仍然不足，经济回升的基础还不稳定，发展也不平衡。同时，我们更要看到面临的重大机遇。一是金融危机引发的产业洗牌，海内外优强企业在全球寻求低成本扩张，南昌作为长江、珠江三角洲及海峡西岸经济区的腹地，无疑是产业转移的首选之地。二是国家继续实施积极的财政政策和适度宽松的货币政策，出台推动经济结构调整和战略性新兴产业发展等具体举措，为我们提供了加快发展的大环境。三是今年“中博会”、明年“七城会”在南昌举办，高铁、地铁的开工建设，将进一步拉大城市的发展空间。四是经过近年来持续“打基础、蓄势能”，支柱产业聚集效益逐步显现。五是鄱阳湖生态经济区规划上升为国家战略，省委、省政府将南昌列为统筹城乡经济社会发展一体化试点城市，为争取政策、项目、投资等各类经济要素的加速聚集，发展低碳生态经济，打造宜居宜业城市，提供了巨大的发展引擎。只要我们审时度势，科学定位，进一步调动广大干部群众的积极性和创造性，团结拼搏，就一定能赢得主动、抢占先机，实现新起点上的新跨越。

　 2010年政府工作的总体要求是：全面贯彻落实党的十七届四中全会、省委十二届十一次全会、市委九届九次全会精神和中央、省、市经济工作会议精神，深入实践科学发展观，按照做好“两篇文章”、“在六个方面走在全省前列”的总体要求，紧紧围绕“企业增效、就业增加、财政增长、百姓增收”目标，积极推进鄱阳湖生态经济区建设，全力实施山江湖综合开发，不断深化改革开放，加快新型工业化和新型城镇化进程，强力推进经济大发展、城乡大变样、文明大提升“三大工程”，全面完成“十一五”规划，为启动实施“十二五”规划，实现南昌赶超进位、跨越发展打下坚实的基础。按照上述总体要求，今年全市经济社会发展的主要预期目标是：

　 地区生产总值增长13%；

　 财政总收入增长13%；

　 地方一般预算收入增长13%；

　 全社会固定资产投资增长32%，力争42%；

　 规模以上工业增加值同口径增长16%；

　 社会消费品零售总额增长16%；

　 实际利用外资增长8%；

　 实际利用内资增长8%；

　 城镇居民年人均可支配收入增长8%；

　 农民年人均纯收入增长9%；

　 万元生产总值能源消耗比上年下降3%；

　 人口自然增长率控制在7.9‰以内；

　 城镇登记失业率控制在4%以内；

　 居民消费价格总水平控制在3%左右。

　 上述预期目标，既体现了对中央和省关于科学发展、快速崛起重要战略部署的落实，也注重了与担当好鄱阳湖生态经济区“排头兵”地位的衔接，更考虑了全面完成“十一五”目标的要求，经过努力是可以完成和超额完成的。

　 为了实现今年政府工作的总体要求和全市经济社会发展的预期目标，我们将采取以下主要工作举措：

　 （一）着力培育优势产业，推动经济结构战略性调整

　 以重大项目为突破口，继续加快先进制造业重要基地、区域性商贸、物流、金融中心的建设，推进一、二、三产业的协调和可持续发展，迅速提高经济质量、壮大经济总量。

　 强力主攻新型工业化。坚持科技强市，坚持以市场为导向，一手推进高新技术改造提升传统产业，一手注重培育战略性新兴产业。坚持质量兴市，加快推进实施标准化战略，发挥国家高新技术产业集群标准化示范基地辐射带动作用。以工业园区为主战场，围绕“一园、两基地、三产业、四重点”，继续抓好“3010”工程和“十百千亿”工程。精心实施科技创新“六个一”工程，主攻光伏材料、清洁汽车及动力电池、航空制造、生物与新医药、半导体照明、现代农业与绿色食品、文化创意、新材料等8项优势高新技术产业，用3年左右时间培育100个创新型企业、实施30项科技重大产业化专项计划、建设5个国家级研发平台、办好6个高新技术产业特色基地、组建30个优势科技创新团队。重点实施南昌航空工业城、江铜铜管二期建设和江铜铜板带续建、江铃股份小蓝生产基地、江西赛维薄膜太阳能电池硅片和电池模组件、南昌印钞厂二期扩建等一批投资10亿元以上的重大项目。采取资产重组、嫁接改造等形式，推进市属改革企业资源整合和股份制改造，优化国有企业产权结构，做强做大昌工控股公司。继续支持企业提高竞争力，着力打造一批优强企业。

　 加快发展高效生态农业。全面落实各项强农惠农政策，继续加大“三农”投入。强力推进土地开发整理，确保耕地占补平衡，提高耕地保护质量。下大力气抓好粮食高产创建、中低产田改造，大力开展农田水利基本建设，加快灌区配套改造，提高水稻耕种收综合机械化水平。加快农业科技创新和先进技术推广，提高农业气象服务和人工影响天气水平，提高农业综合生产能力。深入推进农业产业化“双十双百双千”和“双带提升”工程，继续整合涉农项目，调整农业产业结构，打造一批现代农业标准化示范区、农产品生产基地和特色农业村。大力扶持蒋巷、黄马等现代农业科技示范园形成特色、加快发展。加强标准化畜禽养殖场、养殖小区建设，大力提高畜禽养殖规模化水平。稳步发展渔业和生态绿色产业。完善动物疫病防控体系和农产品质量检验检测体系，确保农产品质量安全。

　 拓展提升现代服务业。全面实施现代服务业发展规划，着力推进“五个十”（十大商贸、十大物流、十大宾馆、十大总部基地、十大旅游项目）建设。一是提升商贸服务水平。进一步提升市级商业中心和红谷滩中央商务区的商贸业态，继续推进城区商业副中心建设。加快推进恒茂梦时代广场、铜锣湾商业广场、洪城大市场升级改造等一批重大项目建设。继续完善社区商业网点，推进15个农贸市场、深圳农产品批发市场改造升级和“万村千乡”市场农家店信息化工程。抓好“家电下乡”、“汽车下乡”、“粮库退城进郊”工作。二是加快发展现代物流业。加快推进保税物流中心项目、向塘公路铁路物流项目和南昌华南工业原料物流城建设，启动昌北航空物流中心等一批物流中心建设。三是不断健全金融服务体系。大力推进红谷滩金融大街建设，继续引进各类金融机构，鼓励金融产品创新。积极发展村镇银行和小额贷款公司，切实缓解“三农”和中小企业融资难问题。加强投融资平台、融资担保平台建设，多渠道筹措城建和产业发展资金。培育企业上市，扩大债券发行，大力发展资本市场。四是大力发展旅游、会展产业。落实旅游发展规划，整合优化旅游资源，加强旅游基础设施建设，进一步打响云水滕王阁、八一英雄城、书画八大山人、候鸟天香园、南昌中国傩园等城市旅游品牌。申办第40届（秋季）全国制药机械博览会、全国（秋季）农机产品订货交易会，办好中国绿色食品博览会、中国南昌国际汽车展、绳金塔庙会、八大山人文化产业博览会。五是大力发展服务外包产业。落实服务外包扶持政策，引进龙头企业，加快江西慧谷—红谷滩创意园、用友南昌软件园、泰豪动漫产业基地建设。加大服务外包人才培训力度，做强南昌“中国服务外包示范城市”的实力。

　 促进非公经济、中小企业发展。进一步营造鼓励全民创业的环境，完善促进非公经济和中小企业发展的政策。加强中小企业信用担保体系建设。继续开展市级中小企业创业基地认定工作，完善各类中小企业公共服务平台，加强中小企业教育培训。吸引和鼓励外来投资者、先富起来的农民、返乡农民工和大中专院校毕业生到小城镇投资办厂、经商创业，形成特色产业，增强中小企业的集聚力。

　 （二）加快打造现代中心城市，统筹城乡协调发展

　 围绕“山江湖”综合开发新格局和推进新型城镇化建设的要求，坚持核心区与各组团并重、老城区与新城区并重、城市与乡村并重，不断强化规划引导、完善城市功能、提升城市品位。

　 统筹城乡规划。完成全市经济和社会发展“十二五”规划编制工作。抓紧《南昌市城市总体规划》规划成果报批；抓紧完成市县两级土地利用总体规划修编的报批。以城市总体规划为统领，围绕鄱阳湖生态经济区建设目标，以产业分布和功能定位为基础，完成“山江湖”发展战略城市规划的重点布局规划，加快启动瑶湖、九龙湖、湾里、乐化、莲塘等组团控规编制，完成马兰圩周边地区控规编制。完成中心城区控制性详细规划100%覆盖和《南昌市近期建设规划（2011—2015）》的编制工作。重视发挥轨道交通引领城市规划建设的作用，编制完善沿线用地控制性详细规划。推进村镇建设规划和污水管网建设规划的编制。继续做好南昌市历史文化名城保护、区域交通、象湖西岸、服务外包产业发展等专项规划的编制。严格规划管理，保证规划执行的权威性和严肃性。

　 统筹城乡基础设施建设。全力支持国家和省重大重点工程建设。完成瑶湖大桥扩建与麻丘互通立交改造、昌九城际铁路、前湖互通立交等项目建设，加快推进昌北国际机场扩建、轨道交通、火车站东广场改造、昌东大道、昌南大道延伸、青山湖大道改造、云湾公路、红湾公路、艾溪湖大桥、滨江规划路网及“五站一中心”、城市客运立体停车场等工程建设。继续加快红谷滩新区建设步伐和朝阳片区成片开发，启动实施老城区低洼危旧房和青山湖大道沿线城中村改造。加快南昌国际体育中心、湾里射击中心等项目建设。续建南昌市城市天然气管网工程、泉岭垃圾焚烧发电厂、南昌市城市供水应急水源工程等项目。加快朝阳公园等公共设施建设。继续实施农村饮水安全工程、病险水库除险加固工程，加快推进鄱阳湖治理二期工程建设。加大农村渡改桥、农村电网完善等建设力度，建设通自然村水泥公路1000公里，启动农村公路危桥改造工程；推进农村中小学危房改造及卫生院、敬老院改扩建，提升农村公共服务能力。以新型城镇化和百强示范镇建设为契机，抓好瑶湖、象湖、九龙湖、湾里、乐化等5个组团城镇，莲塘、长堎、民和、龙津等4个城关镇及第一批特色建制镇建设。

　 统筹城乡生态环境营造。全面推进“森林城乡、花园南昌”建设，大力实施“五大都市森林公园”、“五条都市绿色长廊”、“五大森林版块”、“五大城郊生态绿化工程”生态建设。健全完善污染监测工作，加大面源污染治理力度，强化水资源保护，加快朝阳洲片区水系、乌沙河、艾溪湖流域幸福渠等综合整治和工业园区污水收集管网系统建设。启动赣江市民公园三期、玉带河景观提升工程。突出重点行业、重点领域的节能减排工作，全面完成“十一五”污染减排目标。按照“户集、村收、乡镇运、县区处理”的模式，继续推进乡村清洁工程试点。

　 统筹推进城镇精细化管理。全力抓好全国文明城市、全国“双拥模范城”七连冠、无障碍城市创建，扎实做好迎接国家卫生城市复检工作。按照重心下移的原则和“统一监督、两级指挥、四级联动、全面履责”的工作模式，加快建立专业对口、责权统一、高效有序的城市管理机制。制定完善公共绿地、景观照明、市政设施改造、户外广告设置等城市专项管理办法。继续开展以“和谐交通、文明出行”为主题的道路畅通工程，进一步完善老城区交通干道、支路系统，千方百计增加停车场点，强化交通日常监管，提高现有道路通行能力。加快推进南昌数字城管建设，完成“南昌市城市管理数字化信息系统”平台开发，突出交通秩序、市容市貌、文明素质等重点，大力开展店招店面、背街小巷、占道摊点、集贸市场、城乡结合部、车辆停放、非法营运等专项整治，确保整洁有序。

　 （三）继续深化改革开放，构建科学发展新机制

　　 坚持大开放主战略，做好改革大文章，消除发展障碍，创新体制机制，增强发展的活力和动力。

　　 加快推进各项改革。一是全面完成市县政府机构改革和事业单位岗位设置，稳妥推进事业单位绩效工资改革；继续深化乡镇机构改革，调整事业单位存量；逐步建立健全劳动、资本、技术、管理等生产要素与贡献挂钩的分配制度，完善高层次人才激励政策。二是完成省属下放国有工业企业和市属国有工业企业改革，不断健全国有资产监管体系。有序推进农垦、粮食、农业、水利、林业、商贸流通、交通等七项国有企业改革。三是全面推进医药卫生体制改革。将医疗保险范围进一步扩大到国有及国有控股关闭破产企业、困难农林水企事业单位、困难农垦企业和城镇困难大集体企业职工，适当增加保险补助。四是全面推进财政改革，深化预算管理、公务支出、资本收益、财政监管等制度改革，进一步培植财源，夯实财政基础。五是稳定和完善农村基本经营制度，规范土地承包经营权流转，保障农民的土地承包经营权。坚持和落实最严格的耕地保护制度和最严格的节约用地制度，完善农村宅基地制度，严格农村集体建设用地的管理。继续深化集体林权制度改革。六是推进交通运输体系改革，整合交通运输资源，加快传统运输产业向现代运输服务业转型。七是进一步完善和规范政府统一规划、统一储备、统一出让和统一监管的土地市场管理机制。科学编制年度土地供应计划，严格落实经营性用地和工业用地招拍挂公开出让制度，强化土地供后监管，加大闲置土地清理处置力度。

　　 加大力度招商引资。进一步开阔视野和思路，不断充实重大招商项目库。突出重点区域、重点企业，依托各类园区或载体，坚持走出去、请进来，注重以商招商、产业招商、小分队招商和会展招商，重点引进一批技术含量高、带动能力强、产业链条长、经济效益好、就业容量大的项目。精心办好中国中部投资贸易博览会、金秋经贸活动月等重大招商引资活动。坚持亲商、安商、富商，关心在昌投资的各类企业发展，主动解决项目推进中遇到的各类重大问题。转变外贸增长方式，完善外贸出口预警机制，建立重点进出口企业无障碍联系机制，优化出口产品结构，力争出口稳定增长。

　 加强对外合作交流。积极参与长三角、珠三角、海峡西岸经济区、鄱阳湖生态经济区等区域性经济合作，加快产业对接、项目对接步伐。重点加强与中央企业、世界500强、国内200强的交流与合作，扩大合作领域，创新合作机制，提升合作层次。加密南昌至台北直航航班，不断拓展昌台经贸关系。支持有条件的企业到境外设立贸易网点，建立生产基地。

　 （四）深入实施民生工程，不断提高人民群众的满意度

　 认真落实国家和省出台的各项政策，坚持新增财力和公共资源向社会民生倾斜，不断加大保障和改善民生工作力度，今年重点围绕解决涉及群众切身利益的问题，实施好八个方面的民生工程。

　 一是千方百计扩大就业促进创业。落实有利于就业的扶持政策，搞好就业帮扶、技能培训、创业扶持，推进高校毕业生、困难就业群体和农村劳动力就业。重点是新增城镇就业7.27万人，新增“4050”人员就业0.69万人，零就业家庭就业安置率100%，残疾人就业率达66%以上；新增转移农村劳动力3.2万人；完成小额担保贷款1.8亿元。

　　 二是健全社会保障体系。扩大个体工商户、非公有制企业从业人员和灵活就业人员、城镇大集体职工参加养老保险。重点是城镇职工参加养老保险104.5万人、参加失业保险56.64万人、参加工伤保险63万人；新型农村社会养老保险参保16万人；农民工参加工伤保险12万人。提高义务兵优待标准。

　　 三是提高社会救助水平。重点是逐步提高城乡低保对象补助标准，提高农村五保供养标准，提高20世纪60年代精简退职老弱残职工救济补助标准，提高城镇大集体困难企业未参保退休职工、农林水困难企事业单位未参保退休职工、手工业联社大集体未参保退休职工生活补助；比照城镇大集体困难企业未参保退休职工生活补助标准，提高城镇“三无”特困群众供养标准，完善城乡临时救助。实行计划生育奖励制度；建立因自然灾害死亡人员抚慰金制度。进一步完善基层司法法律援助体系。

　 四是加大医疗保障力度。重点是继续实施“光明·微笑”工程；扩大城镇职工基本医疗保险覆盖范围；推进新型农村合作医疗制度建设；落实农村医疗救助配套资金、城市医疗救助配套资金；免费提供社区公共服务卡；完善城乡困难群众大病医疗救助制度；扩大儿童一类疫苗接种范围；完善优抚对象看病就医制度；建立乡镇卫生人员经费保障机制；建设药品实时监控系统。

　 五是提升教育保障和公共文化福利水平。重点是继续实施和完善“奖贷补助免相结合，大中小学校全覆盖”的济困助学政策；开展就读农村中职免费教育试点；设立贫困家庭学生高考入学政府资助金制度；建设一批乡镇文化站、农家书屋、“村村通”广播电视；免费开放博物馆和纪念馆；加强基层体育健身场所建设。

　 六是强化住房保障工作。重点是建设廉租住房5000套、25万平方米；经济适用住房1500套、10万平方米，全部采用货币补贴方式进行保障；做好农村困难群众维修住房工作；改造城市和国有工矿棚户区399.73万平方米。

　 七是加大扶贫开发力度。重点抓好整村推进扶贫开发；在70个扶贫开发工作重点村实施村级扶贫规划；落实产业化扶贫项目贷款贴息资金。

　 八是改善人民生产生活条件。重点推进660个社会主义新农村村点建设；建设农村户用沼气池2000户；建设农村无害化户厕1.87万座；推进区域性乡镇垃圾无害化填埋场建设；扩大农业政策性保险试点和农机具补贴范围。

　 （五）大力发展各项事业，不断提升社会进步水平

优先发展教育事业。保障教育经费投入，实施农村中小学标准化建设，继续优化城市教育网点布局，加强中小学校舍安全工程建设，推进义务教育均衡发展；推进特殊教育学校、优质高中建设。扩大优质教育资源覆盖面。继续实施职业教育实训基地、示范性中等职业学校建设计划。有序发展幼儿教育和农村中小学现代远程教育；加强民办教育的监督和管理。推动各层次教育协调发展。

大力推进科技进步。推进南昌创新型城市建设，继续开展“十城千辆”、“十城万盏”示范工程，力争更多项目得到国家和省高新技术产业等基金支持。完善科技产业规划，实施知识产权战略，建立产业技术创新联盟，抓好科普宣传教育。

　　 继续加大人才工作力度。引进一批急需和重点高层次人才，拓宽海外人才智力引进渠道。落实“211”企业经营管理人才、“521”专业技术人才培养计划。推动博士后工作站建设。

强化公共卫生服务。扩大免费公共卫生服务范围。实施国家基本药物制度和取消药品加成试点。完成市一医院青山路分院改造，加快推进市三医院朝阳院区、市洪都中医院红谷滩院区等项目建设。大力开展爱国卫生运动。

　 促进文体事业繁荣。围绕建设“文化大市、文化强市”目标，大力推进文化体制改革，促进文化事业繁荣和文化产业发展。完成西湖市民文化活动中心和八大山人真迹馆建设，启动市群艺馆、南昌·中国雕塑博览园建设，推出一批特色鲜明、群众喜好的文化精品，重点打造大型情景剧《滕王阁序》，办好首届南昌·中国雕塑艺术节。推进李渡酒业申遗工作。搞好第三次全国文物普查。加强出版物市场和网络监管，保障文化和网络市场健康发展。积极开展群众体育活动，举全市之力做好“七城会”各项筹备和配套工作，全力推进体育场馆建设，确保“数字城运、绿色城运、和谐城运”目标的实施。积极参加第十三届省运会。

　 加强人口计生工作。认真贯彻落实省《人口和计划生育“一票否决”制度实施办法》，严格人口与计划生育目标管理，落实利益导向政策，加大出生人口性别比综合治理，稳定低生育水平，提高人口素质。

　 （六）扎实推进平安南昌建设，营造和谐稳定的发展环境

　 进一步加强社会治安综合治理。深化平安南昌创建，推进“天网工程”建设，构建融打防管控于一体、全面覆盖网上网下的社会治安防控体系。继续加强基层司法所建设，全面试行社区矫正工作。深化公安“三基”工程和信息化、执法规范化、和谐警民关系建设；加强流动人口服务和管理；依法严厉打击刑事犯罪行为，深入开展“打黑除恶”、打击“两抢一盗”、禁毒等专项斗争。

进一步做好信访工作。坚持属地管理、分级负责，健全矛盾纠纷排查化解机制、信访督查督办机制，畅通信访渠道，引导群众理性合法表达利益诉求，积极预防和妥善处置群体性事件。坚持领导信访接待日、市政府领导信访值班和重大信访事项领导包案等制度，强化信访工作的“一岗双责”和对县区、乡镇考核机制，努力把矛盾解决在基层、解决在萌芽状态。

　 进一步全力抓好安全生产。强化“安全生产一刻也不能放松”理念，严格落实安全生产责任制和重特大事故责任追究制，健全各类安全生产制度，毫不松懈抓好生产安全、食品药品安全、交通安全、消防安全、森林防火等重点行业、重点领域的安全生产，深入开展隐患排查整治，坚决杜绝各类重特大安全事故发生。

　 进一步提升应急处置能力。健全指挥统一、反应灵敏、协调有序、运转高效的应急指挥机制，完善细化应急预案，强化应急值守和实战演练，加强应急救援队伍和应急装备建设，推进应急避难场所建设，提高应对极端灾害天气和预防处置各类突发公共事件的能力。

　 面对复杂多变的发展形势和充满前景的发展机遇，全市各级政府更要增强责任感和紧迫感，切实加快职能转变，认真履行自身职责，使政府工作经得起历史、人民和实践的检验。一要加强法治建设。坚持依法行政，全面实施“五五普法”依法治市规划。加强和改进政府立法工作，规范行政执法行为；自觉接受市人大及其常委会的法律监督、工作监督和市政协的民主监督，重视新闻舆论和社会公众监督，支持监察、审计部门依法独立监督。认真做好人大代表议案、建议和政协提案的办理工作，巩固提高代表委员满意率；坚持定期向市人大及其常委会报告工作和向市政协通报情况。密切与各民主党派、工商联、无党派人士和社会各界的联系，发挥好工、青、妇等人民团体的桥梁和纽带作用。二要增强服务意识。按照深入开展“创业服务年”活动的要求，依法加强公务员队伍管理，不断提高政府的公信力和执行力，进一步改进机关作风，积极主动地为群众、企业和基层提供优质规范服务。多谋惠民之策、多办利民之事。三要提高工作效能。发扬真抓实干、雷厉风行的作风，对重大决策、重点工作和重大项目，严格执行责任制度和考核问责制度，着力解决不作为、乱作为、慢作为等问题。狠抓“两集中三到位”落实，强化审批监督，规范办事程序，减少办事环节。减少会议和文件，改进会风和文风，腾出更多的精力谋大事，集中更多的时间抓落实。四要坚持廉洁自律。坚持执政为民、从严治政，严格执行领导干部廉洁从政各项规定。进一步推进惩治和预防腐败体系建设，落实党风廉政建设责任制。严格规范规划、土地、工程建设、财政资金使用、政府采购、国有资产转让等公共资源管理权力的运行，严肃查处违法违纪行为，继续开展好专项整治工作，切实纠正损害群众利益的不正之风。坚持厉行节约，严格控制公务消费，努力降低行政成本。

　 各位代表，新世纪的头十年，南昌取得了让世人瞩目的成就；今年是新世纪又一个十年的开局之年，我们肩负的使命更加光荣而艰巨。让我们更加紧密地团结在以胡锦涛同志为总书记的党中央周围，高举中国特色社会主义伟大旗帜，全面落实科学发展观，在省委、省政府和市委的正确领导下，进一步弘扬“大气开放、诚信图强”的城市精神，坚定信心、锐意进取，为实现南昌快速崛起的新跨越，作出新的更大的贡献！
